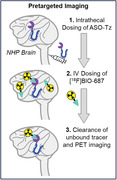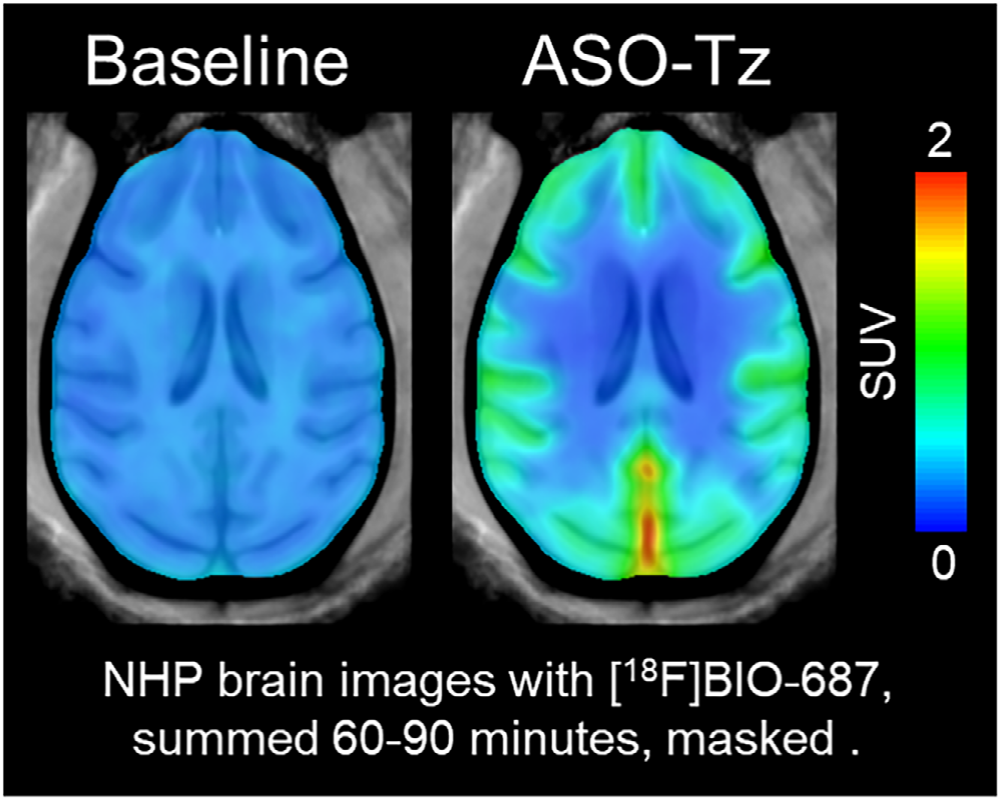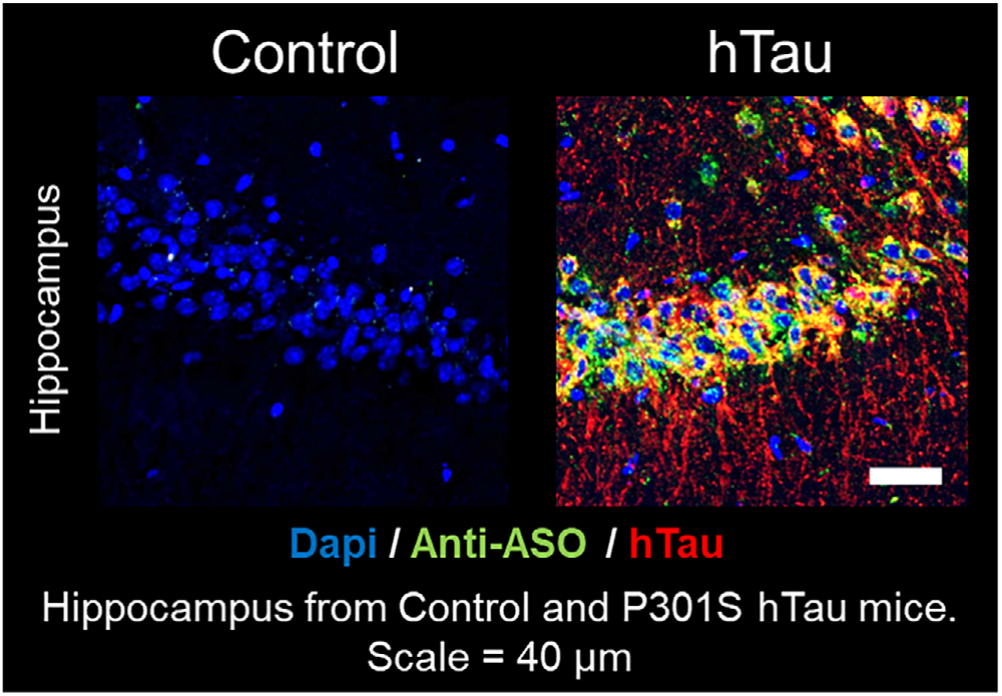# Pretargeted Imaging for In Vivo Assessment of ASO Therapy Delivery in Alzheimer’s Disease

**DOI:** 10.1002/alz.093694

**Published:** 2025-01-09

**Authors:** Brendon E Cook, Thomas C Pickel, Mario Amatruda, Qize Zhang, Sangram Nag, Phillipe Bolduc, Rouaa Beshr, Anton Forsberg Morén, Cathy Muste, Prodip Datta, Piotr Ochniewicz, Yasir Khani Meynaq, Jonathan M. DuBois, Stephanie K Klein, Christer Halldin, Luca Passamonti, Maciej Kaliszczak

**Affiliations:** ^1^ Biogen, Cambridge, MA USA; ^2^ Karolinska Institutet, Stockholm Sweden; ^3^ Ionis Pharmaceuticals, Carlsbad, CA USA

## Abstract

**Background:**

Intrathecally (IT) delivered antisense oligonucleotides (ASOs) are promising therapies that can reduce tau pathology in Alzheimer’s Disease (AD). However, current plasma and CSF sampling methods to estimate brain tissue exposure of ASOs are inherently limited, hampering ASO clinical developmental plans. We developed the PET tracer [18F]BIO‐687, which binds ASO conjugates (ASO‐Tz) in vivo, allowing us to image ASO distribution in a living brain using “pretargeted” imaging. Pretargeted imaging proceeds by: 1) intrathecal ASO‐Tz dosing; 2) brain distribution of the ASO‐Tz; 3) intravenous (IV) [18F]BIO‐687 administration, where tracer binds to ASO‐Tz in the brain via ‘click‐chemistry’ reactions, followed by PET imaging. Pretargeting enables longer imaging timepoints while reducing radiation dose to patients. Using pre‐clinical experiments, we demonstrate the feasibility of pretargeted imaging to assess ASO distribution in AD.

**Method:**

A Malat1 ASO tool compound was developed and conjugated as a surrogate for therapeutic ASOs treating AD. ASO distribution was imaged in a non‐human primates (NHP) experiment. ASO‐Tz was IT dosed (20 mg), followed 24 hours later by IV [18F]BIO‐687 injection and PET imaging. Baseline scans of [18F]BIO‐687 with no ASO‐Tz were also performed. In a separate experiment, the Malat1 ASO‐Tz was dosed IT in an AD mouse model expressing hTau (P301S) and age‐matched controls. One week later, mouse brains were stained for hTau (AT8) and ASO distribution using immunofluorescence.

**Result:**

The PET experiment in NHP showed high [18F]BIO‐687 binding in brain regions where ASO‐Tz distributed, indicating successful “pretargeted” imaging. As expected for any IT‐delivered ASO, uptake was higher in hindbrain and cortex. In mice, ASO signal (identified by immunofluorescence) was significantly higher in the hippocampus of P301S than control mice (p<0.05). Unexpectedly, higher ASO concentration was correlated with higher hTau concentration in regions such as the hippocampus and cortex (p<0.05).

**Conclusion:**

The NHP PET imaging data clearly demonstrated the feasibility and utility of pretargeted imaging for assessing Malat1 ASO (or potentially any ASO) distribution. Concurrently, our results from the P301S mice study intriguingly show that Malat1 ASO distributes more in brain regions showing higher tau pathology. Our findings support further pretargeted ASO imaging studies in healthy volunteers and AD patients.